# Comparative studies of two generations of NanoString nCounter System

**DOI:** 10.1371/journal.pone.0225505

**Published:** 2019-11-21

**Authors:** Lianbo Yu, Sagar Bhayana, Naduparambil K. Jacob, Paolo Fadda

**Affiliations:** 1 Center for Biostatistics, Department of Biomedical Informatics, The Ohio State University, Columbus, Ohio, United States of America; 2 Department of Radiation Oncology, The Ohio State University, Columbus, Ohio, United States of America; 3 Genomics Shared Resource, Comprehensive Cancer Center, The Ohio State University, Columbus, Ohio, United States of America; The University of Tokyo, JAPAN

## Abstract

The NanoString nCounter System has been widely used in basic science and translational science research for the past decade. The System consists of two instruments: the PrepStation and the Digital Analyzer, and both have been continuously improved with evolving technologies. A great amount of research data have been generated at multiple research laboratories with the employment of different generations of the System. With the need of integrating multiple datasets, researchers are interested to know whether signals are comparable between different generations of the System. Toward this end, we designed a profiling study to compare performance of two generations of the NanoString nCounter System using a common set of biological samples. Using graphical tools and statistical models, we found that two different generations of NanoString nCounter System produced equivalent signals and signal deviations are in the range of random background noises for the medium-high expression levels.

## Introduction

The NanoString nCounter System is a technology based on digital hybridization assays that capture and sensitively count nucleic acids and proteins derived from different biological sources, such as tissues, cells and blood lysates, FFPE samples, body fluids, or low numbers of cells [1–4]. The System is designed to measure directly RNA or DNA molecules in a single reaction without amplification, so that polymerase chain reaction (PCR) related biases are avoided: this makes NanoString a valid and reliable platform for both research and clinical applications. The System is constituted by two instruments: the PrepStation and the Digital Analyzer. The PrepStation consists of a multi-channel Robotic Liquid Handler that performs a magnetic bead sample clean-up in order to remove the probe excess that did not bind tagged molecules, and it immobilizes and stretches labelled molecules on the cartridge surface by applying an electric field. The Digital Analyzer, instead, is a multi-channel epifluorescence scanner that detects tagged molecules. Using a CCD camera via a microscope objective lens, the scanner secures data by taking picture of color-coded barcodes present on the cartridge surface per lane. Each cartridge contains 12 separated lanes per 12 samples run. The Images that each cartridge lane generates are converted to RCC (Reporter Code Count) files that contain the target counts per sample lane.

The Genomics Shared Resources at The Ohio State University acquired the first generation of nCounter Max System on 2009 (from now on indicated as “first generation”) and upgraded the platform on 2017 with the nCounter Flex System (from now on indicated as “second generation”). The new Digital analyzer of the nCounter Flex System, with a new light source, provides a more stable illumination power, eliminates high voltage, reduces calibration errors, removes the need of immersion oil, avoids focusing error, and improves the instrument maintenance. To evaluate similarities and differences between the Max and Flex Systems, we conducted a comparative analysis by processing a common set of biological samples with the two generations of NanoString platform. In particular, we used the Human microRNA V3 assay, which detects 800 biologically-relevant human mature microRNAs (miRNAs). Because of the mature microRNAs’ small size (19 to 22 nucleotides), the assay is designed to ligate a DNA tag (miRtag) to each mature microRNA using a ligase enzyme. The tagged microRNA molecules are combined with the Code-set that consists of a multiplexed library of two single-stranded sequence specific DNA molecules, the Capture and Reporter probes. The Capture probe is a complementary target sequence DNA molecule conjugated with Biotin that allows the tagged microRNA-Capture probe-Reporter probe complex to bind on the cartridge surface. The Reporter probe is a complementary target sequence DNA molecule that contains a color-coded barcode that produces the detection signal.

Huge amount of NanoString data have been generated in the past decade by using different generations of NanoString System. Our designed profiling study for comparing performance of two generations of the NanoString System using a common set of biological samples will bridge the gap between the pressing need of integrating multiple datasets across different generations of the NanoString System and lack of comparative studies on these system differences. In this profiling study, cultured human renal primary tubular epithelial cells (HRPTEC, purchased from Lonza; CC-2553) treated with different Gamma radiation doses and untreated cells (used as a control) were processed for NanoString miRNA profiling by using both the first generation System and the second generation System and results were compared. For data analysis, a variety of statistical methods, such as hierarchical clustering, Pearson’s correlation coefficient (PCC), analysis of variance (ANOVA) models, and locally estimated scatterplot smoothing (LOESS), were employed for signal comparison.

In summary, the main purpose of this paper is to compare two generations of NanoString System in order to provide technical insights for researchers who want to integrate multiple datasets across different generations of NanoString System. To accomplish this goal, we develop a systematic and innovative approach for such comparisons including experimental design, data visualization, statistical modeling, and data interpretation. Moving forward, the proposed approach provides a comprehensive framework that will help for implementing other similar comparative studies, e.g. across diverse platforms, multiple assays, and series of research applications.

## Materials and methods

### Cell culture and treatment

Human renal primary tubular epithelial cells (HRPTEC) were purchased from Lonza (CC-2553) and cultured in renal epithelial cell growth medium (Lonza, CC-3191) supplemented with single Quots supplements (Lonza, CC-4127) that includes 0.5% FBS and the growth factors. As cells reached passage number 5 (15 population doublings are allowed), they were irradiated with GammaCell-40 Irradiator, Cs137 source at a dose rate of 0.94 Gy/min (Best Theratronics) in fractions (4 Gy, 4X4 Gy, 4X4X4 Gy). Cells were then harvested for RNA isolation at two time points (Day 7 and Day 14) after irradiation to screen for microRNA responders indicative of activated signaling pathways post-fractionated radiation. Untreated cells at same passage number were used for comparison. Table 1 shows the samples and their corresponding irradiation treatment.

**Table 1:** Irradiation treatment schedule for all 6 HRPTEC samples.

Sample Number	Dose (Gy)	Days
1	Control (untreated)	0
2	4X4 Gy	7
3	4X4X4 Gy	7
4	4 Gy	14
5	4X4 Gy	14
6	4X4X4 Gy	14

### Sample preparation for miRNA profiling

Total RNA (large and small RNAs) was isolated from all samples by miRVana microRNA isolation Kit (AM1560, ThermoFisher Scientific). The RNA isolation protocol was followed as per the manufacturer’s instructions. A clean-up step (Amicon^®^ Ultra centrifugal filter columns C82301, Merck) was added after RNA elution step in order to dilute/exclude any contaminants that might interfere in NanoString profiling. 200 ng of RNA was used for NanoString miRNA profiling (Human V3 miRNA Panel).

### The NanoString nCounter System

Six samples were run in duplicates per cartridge. 200 ng of total RNA were mixed with a Master Mix containing miRNA tags, target specific bridges and spike-ins in order to evaluate the ligation enzyme efficiency (Ligation Positives). Mature microRNAs were bound to sequence specific miRtags using a Ligase Enzyme provided with the Kit and unligated miRtags were removed using a clean-up enzyme. Samples were diluted with 40 μl of water and denatured for 5 minutes at 85°C. 5 μl of product were combined with 20 μl of Reporter Probes in hybridization buffer and 5 μl of Capture probes and incubated at 65°C overnight (18 hours). Probe excess was removed using a two-step magnetic beads based purification method in PrepStation and target/probe complexes were bound on the cartridge for data collection. The Digital Analyzer collected the data by taking images of immobilized fluorescent reporters in the sample cartridge with a CCD camera. Each cartridge was scanned using 600 fields of view (FOV) with the first generation System scanner and 320 FOV with the second generation System scanner.

### Experimental design

**Fig 1:**
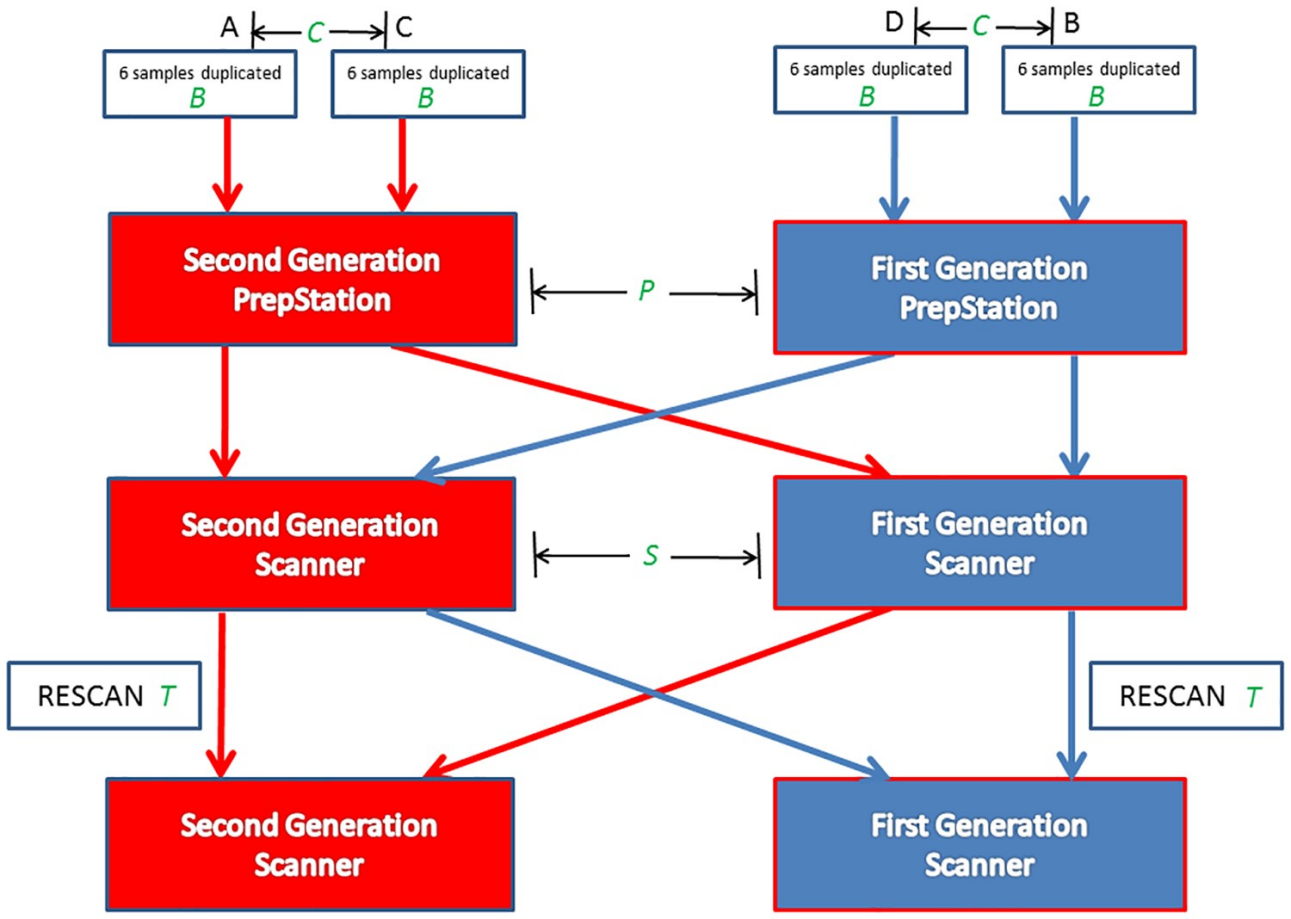
Diagram of experiment design. Four cartridges (A, B, C, D) were used to profile miRNA expression of 6 duplicated biological samples. Two RNA preparation methods and two scanners were combined for different cartridges. Effects in ANOVA models are labeled in green color.

Experiment layout are shown in the diagram in Fig 1. A total of four NanoString cartridges were processed using two generations of the NanoString nCounter System. Six samples were duplicated in each cartridge. The first cartridge (A) was processed using the second generation System and was scanned twice at 320 FOV. Similarly, the second cartridge (B) was processed using the first generation System and was scanned twice at 600 FOV. The third cartridge (C) was processed using the PrepStation from the second generation System and scanned twice with the scanner from the first generation System at 600 FOV. And finally the last cartridge (D) was processed using the PrepStation from first generation System and scanned twice with the scanner from the second generation System at 320 FOV.

### NanoString data analysis

Positive controls (spiked by the NanoString Company in the Code-set) were used for correcting assay efficiency. Negative controls were used to filter out microRNAs with expression at noise level. Median normalization was performed to normalize across samples using all housekeeping genes. Heat-maps were used for data visualization. Pearson’s correlations and scatter plots were used for signal comparison between cartridges.

### Variance decomposition

ANOVA methods have been routinely used to analyze differences in outcomes in the analysis of comparative experiments and clinical trials [5–6]. Under a typical ANOVA model, total variability is partitioned into its component parts, and mean square is calculated to represent variability of the component parts. In this study, we employ three different ANOVA models (at the first time scan, at the second time scan, and across both time scans) on expression values of the 6 HRPTEC samples in each of the 4 cartridges to estimate the sources of variability (i.e. PrepStation, Scanner, Sample, Cartridge, Scan time) at each microRNA. The square root of mean square for each source, denoted as root-mean-square (RMS), is then smoothed over the range of expression levels of all microRNAs by using the LOESS method [7–8]. The fitted LOESS curves of all sources are plotted altogether against the range of expression levels, and then used for comparison among all sources (i.e. PrepStation, Scanner, Sample, Cartridge, Scan time). Further, inference on the differences between two generations of NanoString System is made in relative to the randomness of technical replicates (within- and between-cartridge) and the biological difference.

ANOVA model for a microRNA at the first time scan is

Y~μ+P+S+B+ϵ,

where *Y* is a vector of expression levels, μ represents the overall mean, *P* represents the effect of the PrepStation, *S* represents the effect of the scanner, *B* represents the effect of biological samples, and *ϵ* represents random errors following a normal distribution *N*(0, *σ*^2^).

ANOVA model for a microRNA at the second time scan is

Y~μ+PS+C+B+ϵ,

where *Y* is a vector of expression levels, μ represents the overall mean, *PS* represents the effect of the sum of the PrepStation and the scanner, *C* represents the effect of cartridge, *B* represents the effect of biological samples, and *ϵ* represents random errors following a normal distribution *N*(0, *σ*^2^).

ANOVA model for a microRNA across both time scans is

Y~μ+PS+T+B+ϵ,

where *Y* is a vector of expression levels, μ represents the overall mean, *PS* represents the effect of the sum of the PrepStation and the scanner, *T* represents the effect of scan time, *B* represents the effect of biological samples, and *ϵ* represents random errors following a normal distribution *N*(0, *σ*^2^).

## Results

A total of four cartridges were used for assaying microRNA expression of the 6 biological samples, in duplicate with 12 samples total and applied to each cartridge. Two PrepStation instruments (from first and second generation System) were used for sample preparation, and two scanners (associated to the first and second generation Systems) were used for miRNA expression detection (Fig 1). In order to evaluate similarities and differences between old and new instruments, we performed three separate analyses for expression signal comparison among the four cartridges. First, to evaluate both the effect of PrepStation and Scanner we used expression data at the first time scan to estimate the sources of variation (PrepStation, scanner, biological samples, and residual errors) and compared them over the range of expression levels. Second, to evaluate both the effects of cartridges and the combined effect of the PrepStation and the scanner, we used expression data at the second time scan to estimate the sources of variation (PrepStation & scanner, cartridges, biological samples, and residual errors) and compared them over the range of expression levels. Third, to evaluate both the effects of scan time and the combined effect of the PrepStation and the scanner, we used expression data of cartridge A and B at both scans to estimate the sources of variation (PrepStation & scanner, scan time, biological samples, and residual errors) and compared them over the range of expression levels. For all three analyses, the source of variation from residual errors was estimated from within-cartridge duplicates.

### Heat-map and hierarchical clustering

**Fig 2:**
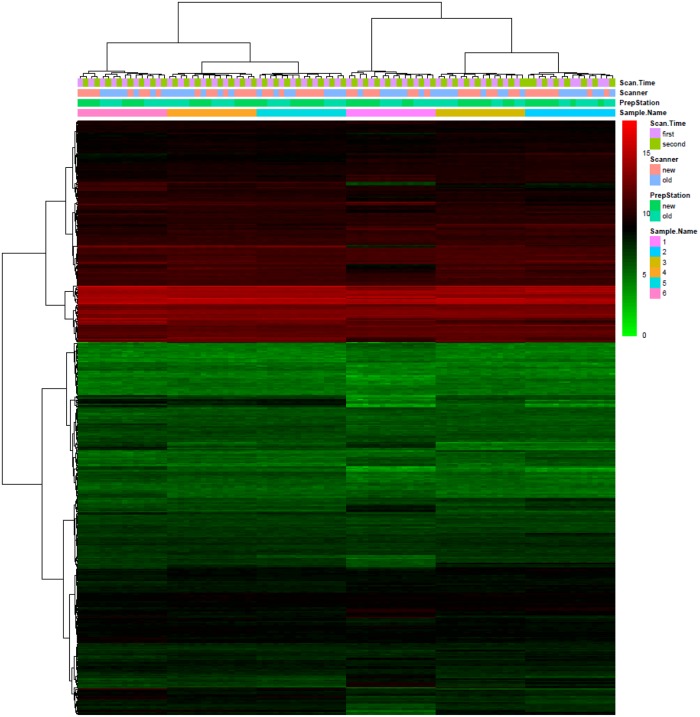
Heat-map of all cartridges. Signal intensity is in log2 scale, red represents higher expression levels, green represents lower expression levels, and black represents medium expression levels. Hierarchical clustering dendrograms are drawn on the heat-map for both microRNAs and biological samples. Samples and cartridges information were listed in legends.

Heat-map of miRNA expression of 12 samples in four cartridges scanned twice by two scanners is displayed in Fig 2. Two-way hierarchical clustering was performed on both microRNAs (rows) and biological samples (columns). Technical replicates of each biological sample are consistently clustered together. Signals within clusters are similar and in contrast the patterns between clusters are clearly different.

### Comparison at the first time scan

PCC of expression levels between each pair of cartridges were calculated separately for each of 12 samples. The range of PCC between cartridge A and B is 0.9926–0.9970; the range of PCC between cartridge A and C is 0.9920–0.9963; the range of PCC between cartridge A and D is 0.9937–0.9970; the range of PCC between cartridge B and C is 0.9938–0.9975; the range of PCC between cartridge B and D is 0.9945–0.9965; the range of PCC between cartridge C and D is 0.9932–0.9968. All PCC are above 0.99, which demonstrates the extreme consistency between technical replicates.

**Fig 3:**
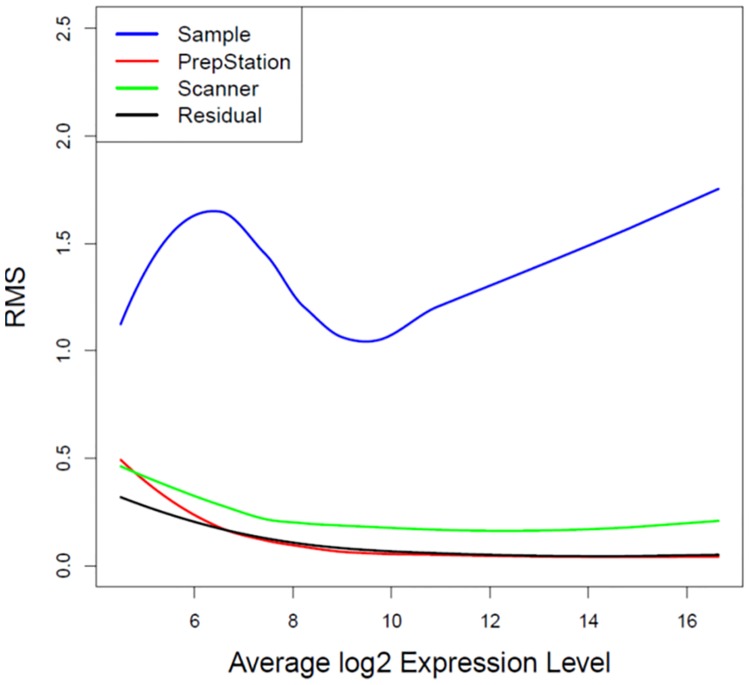
LOESS curve fitting of RMS against average expression levels for the comparison at the first time scan. Variance components of ANOVA models were smoothed over average log2 expression levels through the LOESS fit. Blue curve represents variability of biological samples. Red curve represents variability of PrepStation. Green curve represents variability of scanners. Black curve represents variability of residual errors.

ANOVA model (1) was applied and sources of variation were obtained for each microRNA molecule. Fig 3 shows the fitted LOESS smoothing curves of RMS over the range of expression levels. The RMS of biological samples is far larger than those of other three sources. The RMS of the scanners is a little larger than that of residual errors for within-cartridge duplicates, while the RMS of the PrepStation is basically identical to that of residual errors at the medium-high expression levels. This result demonstrates that the two generation of PrepStations do not provide extra source of variation at the medium-high expression levels, while the differences between the two scanner versions are minor.

### Comparison at the second time scan

PCC of expression levels between each pair of cartridges were calculated separately for each of 12 samples. The range of PCC between cartridge A and B is 0.9926–0.9970; the range of PCC between cartridge A and C is 0.9920–0.9963; the range of PCC between cartridge A and D is 0.9937–0.9970; the range of PCC between cartridge B and C is 0.9938–0.9975; the range of PCC between cartridge B and D is 0.9945–0.9965; the range of PCC between cartridge C and D is 0.9932–0.9968. All PCC are above 0.99, which demonstrates the extreme consistency between technical replicates.

**Fig 4:**
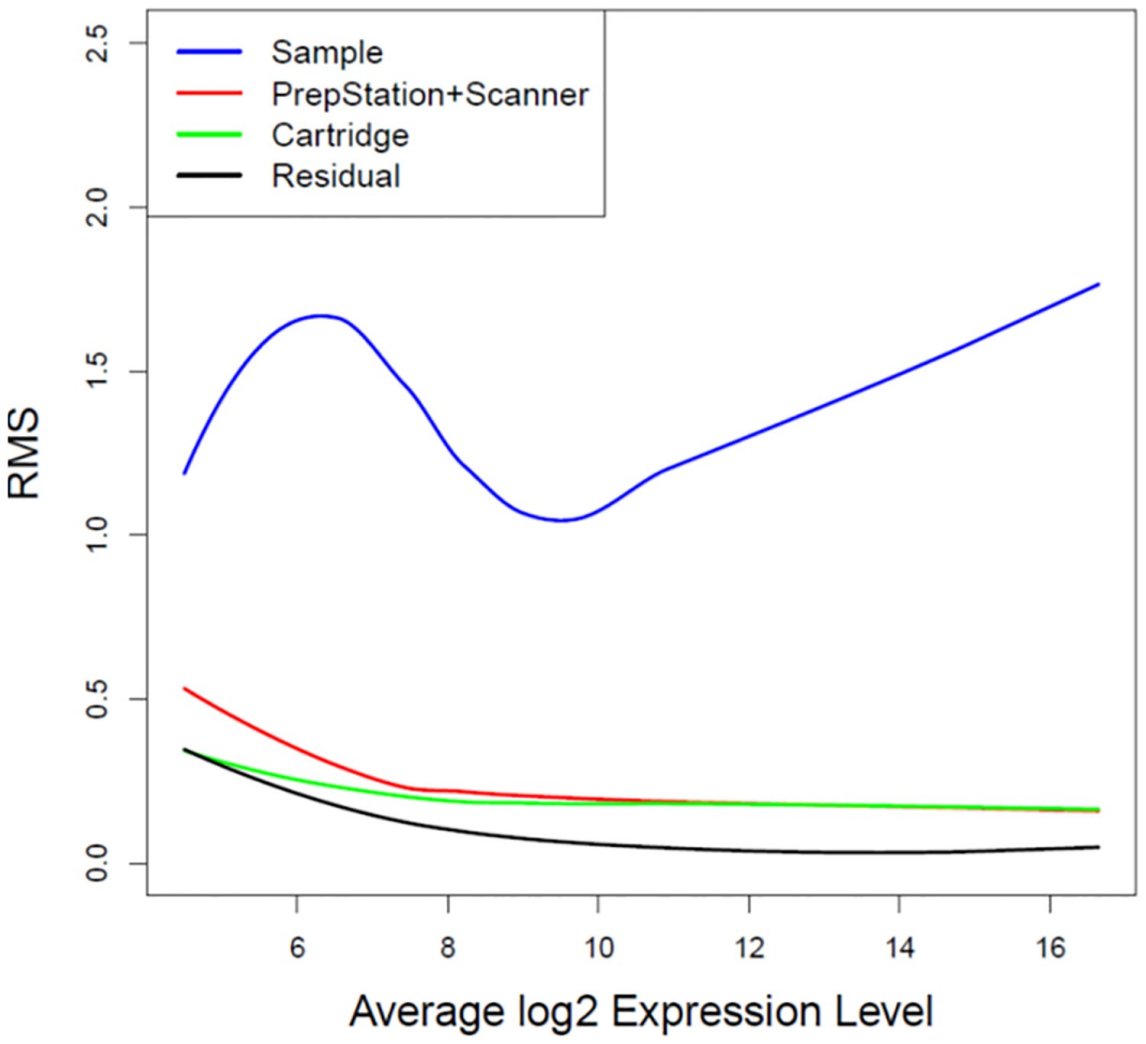
LOESS curve fitting of RMS against average expression levels for the comparison at the second time scan. Variance components of ANOVA models were smoothed over average log2 expression levels through LOESS fit. Blue curve represents variability of biological samples. Red curve represents variability of PrepStation and scanners. Green curve represents variability of cartridges. Black curve represents variability of residual errors.

ANOVA model (2) was applied and sources of variation were obtained for each microRNA molecule. Similarly, Fig 4 shows the fitted LOESS smoothing curves of RMS over the range of expression levels. As expected, the RMS of biological samples is outstanding from the others. The RMS of the combination of preparation and scanners overlaps with the RMS of between-cartridge replicates for medium-high expressed microRNAs. This result demonstrates that the differences between the combination of first generation System PrepStation and its scanner and the combination of the more recent second generation System PrepStation and its scanner are just inseparable from between-cartridge differences for the medium-high expression levels.

### Comparison across both time scans

PCC of microRNA expression levels between each pair of cartridges were calculated separately for each of 12 samples. The range of PCC between cartridge A and B at the first scan is 0.9926–0.9970; the range of PCC between cartridge A and B at the second scan is 0.9923–0.9965; the range of PCC between the first and the second scans for cartridge A is 0.9988–0.9994; the range of PCC between the first and the second scans for cartridge B is 0.9979–0.9990. All PCC are above 0.99, which demonstrates the extreme consistency between technical replicates.

**Fig 5:**
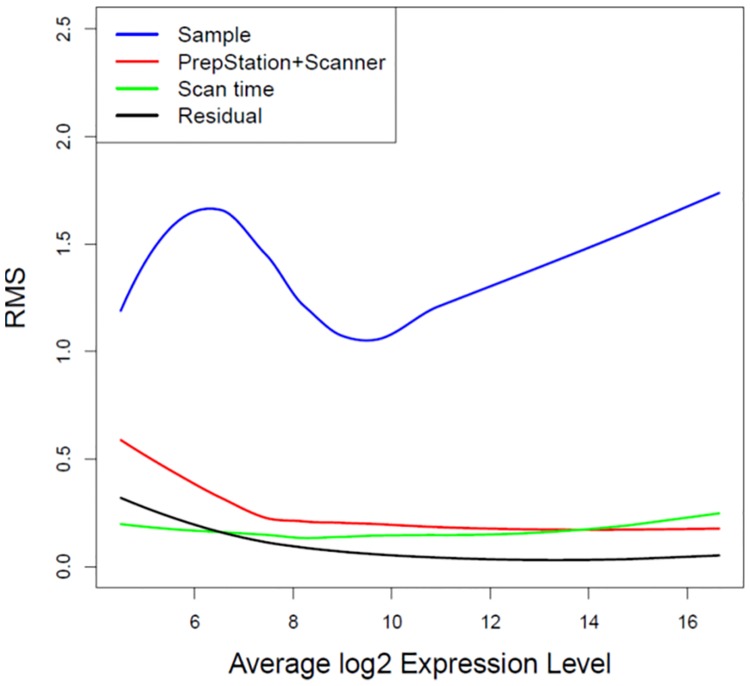
LOESS curve fitting of RMS against average expression levels for the comparison across both time scans. Variance components of ANOVA models were smoothed over average log2 expression levels through the LOESS fit. Blue curve represents variability of biological samples. Red curve represents variability of PrepStation and scanners. Green curve represents variability of scan times. Black curve represents variability of residual errors.

ANOVA model (3) is applied to both scans of cartridge A and B for each microRNA molecule. The fitted LOESS smoothing curves of RMS over the range of expression levels were plotted in Fig 5. The curve for biological samples is the highest among all 4 curves as expected, and the RMS of the sum of the PrepStation and the scanner is in a similar range of the RMS of between-scan replicates for medium-high expressed microRNAs. This result indicates that the differences between the combination of the older first generation System PrepStation and its scanner and the combination of the more recent second generation System PrepStation and its scanner are just irrelevant for the medium-high expression levels since they are at the same level of differences between repeated scans of the same cartridges.

## Discussion

Heat-map and hierarchical clustering demonstrates that there are no systemic biases between old and new PrepStation, between old and new scanners, or even between different combinations of two PrepStation and two scanners. In addition, we performed pair comparisons between cartridges using ANOVA methods to further evaluate differences due to each specific effect of the PrepStation, the scanner, cartridge, or scan time (e.g., comparison between A and D or B and C for the effect of the PrepStation at the first time scan; comparison between A and C or B and D for the effect of the cartridges at the second time scan). All PCCs between each pair of cartridges for each sample are above 0.99, which further support the evidence shown in the heat-map.

ANOVA models allow us to decompose sources of variation and mean square provides unbiased variance estimates for each source. Since ANOVA models are fitted at a single miRNA level, the obtained RMSs are then smoothed over the range of expression levels of all microRNAs. From the first time scan, it shows that the variability due to the PrepStation itself is as similar as the random error for within-cartridge duplicates for most microRNAs, so we can conclude there is no difference between old PrepStation and new PrepStation except at the really low expression levels. Further from the second time scan, it shows that the variability due to the sum of the PrepStation and the scanner is as similar as variability due to difference cartridges except for the low expression microRNAs. Because different cartridges were used for the combination of old PrepStation and old scanner and the combination of new PrepStation and new scanner, the variability of the sum of the PrepStation and the scanner includes the variability of cartridges. Therefore we can conclude that there is no difference between the combination of first generation System PrepStation and its scanner and the combination of the more recent second generation System PrepStation and its scanner for the medium-high expressed microRNAs. However, there could be minor technical differences at the low expression levels, but these technical differences are very small compared to variability between the 6 biological samples. To investigate whether these technical differences at the low expression levels has any impact on comparative analyses between the biological samples, we performed a differential expression analysis between day 7 and day 14 with 5 biological samples for each cartridge. We observe that the proportions of consistent microRNAs in the significance call (differential or non-differential expression between day 7 and day 14) are equivalent for any two cartridges across two systems (i.e. A vs B, A vs D, C vs B, C vs D) and within each system (i.e. A vs C, B vs D) at either low or medium-high expression levels. Therefore we conclude that the variability difference at the low expression levels as showed in Figs 3–5 does not impact comparative analyses.

The comparative results between two generations of NanoString System represent the technical reproducibility and comparability, and they are not dependent on cell line or types of sample used for the following reasons. First, the proposed comparative approach is to compare technical differences at each cell line sample; Second, the biological differences in the 6 cell line samples are only used as a reference. Even though biology variability is different for various cell lines or types of sample, it does not impact the conclusion; Third, many published studies on evaluating NanoString System reproducibility show that technical replicates have great consistency by using various types of cell lines and clinical samples [9–11]. Further, Veldman-Jones *et al*. compared both technical and biologic replicates and concluded that biological variability is far greater than technical variability [11]; Fourth, for the 6 cell line samples we used, we chose control as well as the irradiated samples (from low to high dose). This treatment itself is harsh enough that any technical variability would have been blown out by biological variability.

The comparative approach presented in this study is in particular proposed for comparing two generations of NanoString System. But it can be adapted to other platform comparison (not only different generations of the same platform, but also between different platforms), and also can be easily extended to accommodate more complex designs. We have applied this comparative approach to a FDA Sequencing Quality Control Project (SEQC). In one of its subseries, RNA-Seq experiments were performed by multiple platforms (Illumina HiSeq and Life Technologies SOLiD) at multiple laboratory sites with multiple libraries and FlowCells using four common reference RNA samples. We used the expression data deposited in GEO (accession number GSE47774) to evaluate differences between platforms, between laboratory sites, between libraries, and between FlowCells. Since this application on SEQC data is not the focus of this study, we summarize our findings here that there is a large difference between sequencing platforms, minor difference between laboratory sites, minimal differences between libraries, and no difference between FlowCells.

## Supporting information

S1 File Log 2 normalized data of all cartridges. Nanostring data from all 4 cartridges scanned twice were filtered and normalized as described in ‘**Materials and Methods**’ section. Log2 normalized data is presented, where miRNAs are in rows and samples are in columns with labels for sample number, cartridge name, and scan time.
(TXT)
